# Indoor Air Pollution Aggravates Symptoms of Atopic Dermatitis in Children

**DOI:** 10.1371/journal.pone.0119501

**Published:** 2015-03-17

**Authors:** Eun-Hye Kim, Soyeon Kim, Jung Hyun Lee, Jihyun Kim, Youngshin Han, Young-Min Kim, Gyo-Boong Kim, Kweon Jung, Hae-Kwan Cheong, Kangmo Ahn

**Affiliations:** 1 Department of Social and Preventive Medicine, Sungkyunkwan University School of Medicine, Suwon, Korea; 2 Samsung Biomedical Research Institute, Sungkyunkwan University School of Medicine, Seoul, Korea; 3 Department of Pediatrics, Samsung Medical Center, Sungkyunkwan University School of Medicine, Seoul, Korea; 4 Environmental Health Centre for Atopic Diseases, Samsung Medical Center, Seoul, Korea; 5 Seoul Research Institute of Public Health and Environment, Seoul, Korea; Peking University, CHINA

## Abstract

Most of researches on the impact of indoor air pollutants on atopic dermatitis (AD) have been based upon animal models, in vitro experiments and case-control studies. However, human data to elucidate the role of indoor air pollution on worsening symptoms of pre-existing AD from a longitudinal study are scarce. The objective of this prospective study was to evaluate the effect of indoor air pollution on AD symptoms in children. We surveyed 30 children with AD in a day-care centre, which moved to a new building during the study. These children stayed there for 8 hours a day Monday through Friday, and their daily symptom scores were recorded. Indoor and outdoor air pollutant levels were continuously measured 24 hours a day for 12 months (Period 1 to 4). Data were analyzed using a generalized linear mixed model. Compared to the period before moving (Period 1), concentrations of indoor air pollutants mostly increased after moving (Period 2) and decreased by natural ventilation and bake-out (Periods 3 and 4). The rate of positive AD symptom increased from 32.8% (Period 1) up to 43.8% (Period 2) and 50.5% (Period 3), then decreased to 35.4% in Period 4 (P < 0.0001). When the delayed effects of indoor air pollutants on AD symptoms 2 days later were evaluated, AD symptoms significantly increased by 12.7% (95% CI: -0.01 to 27.1) as toluene levels increased by 1 ppb (P = 0.05). In conclusion, indoor air pollutants increase the risk of AD aggravation in children and toluene in the indoor environment might act as an aggravating factor.

## Introduction

Indoor environment is an important issue for human health. With socioeconomic development, more attention is being paid to indoor air pollution because people spend most of their time indoors, both at home and at work. In a condition, so-called ‘sick building syndrome’ (SBS), patients suffer from nonspecific symptoms of the eyes, skin, and upper airways as well as headache and fatigue [1,2]. Various environmental factors are responsible for SBS, including indoor mold and chemicals originating from building materials and consumer products [3–5]. In particular, children are more susceptible to indoor environmental hazards in many aspects. Compared to adults, the breathing zone for children is closer to the floor where they are more easily exposed to chemicals. Children are biologically vulnerable to the environment as they have higher metabolic rates and minute ventilation relative to their body size, leading to greater exposure to air pollutants [6,7]. In addition, newborns and infants have a larger surface area-to-body mass ratio and their skin may have different absorptive properties compared to adults [8,9].

Many studies have been done to identify the hazardous effects of air pollution on allergic diseases such as asthma, allergic rhinitis, and atopic dermatitis (AD). Since inhalation is the major pathway for air pollutants to affect the human body, many studies have investigated the relationship between asthma and indoor air quality. Exposure to particulate matter with a diameter of 2.5 μm or less (PM_2.5_) in school classrooms is associated with elevated levels of fractional exhaled nitric oxide (FeNO) in asthmatic children [10]. Short-term exposure to nitric dioxide (NO_2_) is significantly associated with wheezing in children with asthma [11]. Environmental tobacco smoke (ETS) exposure during the perinatal period is linked to asthma development [12,13]. There is some convincing evidence that formaldehyde or volatile organic compounds (VOCs) affect asthma symptoms [14]. The mechanism is not fully understood, but many air pollutants are known to form reactive oxygen species (ROSs) and exert their detrimental effect by causing oxidative stress in cells and tissues [15].

AD is a chronic inflammatory skin disorder with severe pruritus. This disease should be managed with appropriate skin care, reduction of inflammation by topical or systemic medication, and strict avoidance of various aggravating factors. Therefore, identification of the aggravating factors for each individual is very important for proper management. Air pollutants such as nitrogen oxides (NO_x_), particulate matters, VOCs and butyl benzyl phthalate (BBzP) seem to act as risk factors in AD [16,17]. However, most of researches into the effect of air pollution on AD have used animal models, *in vitro* experiments and case-control studies in the study design. Human data to elucidate the role of indoor air pollution on worsening symptoms of pre-existing AD from a longitudinal study are still scarce. The objective of this prospective study was to evaluate the effect of indoor air quality on AD symptoms by daily recording of symptom scores and continuous monitoring of indoor air pollutant levels 24 hours a day for 12 months.

## Materials and Methods

### Study population and clinical evaluation

This study was carried out in a day-care center in the northeast region of Seoul between May 2009 and April 2010. This day-care center is located 150 m from a road with heavy traffic, a metro station, and a local market. Among 76 children between the ages of 2 and 7 years, 30 children with AD were enrolled for this study. The diagnosis of AD was determined according to Hanifin and Rajka criteria [18], and the severity was assessed using the SCORing Atopic Dermatitis (SCORAD) [19]. The children’s mean age at the time of enrolment was 4.4±1.2 years, and SCORAD at the initial visit was 11.2±13.7. Their duration of stay in the day-care center was 8 hours a day Monday through Friday. Parents were instructed to bathe their children once daily and to apply moisturizers frequently during the study period. Intermittent use of low potency topical corticosteroids (TCS) and oral antihistamines were employed as needed base.

In June 2009, the center moved into a new building that is located in an area with less traffic and no nearby major sources of pollution, such as factories or incinerators. After moving, bake-out by heating and ventilating the indoor air was performed to remove air pollutants eight times between August and October of 2009. During this period, indoor temperature was kept at 35°C for 6–8 hours during the night. The following day the building was ventilated for 12–18 hours during daytime. Bake-out was done every weekend when the children were absent. In the present study, the research period was divided into four distinct time periods as follows: Period 1, the period before moving (from 20 May 2009 to 14 June 2009); Period 2, the first six weeks with natural ventilation alone after moving into the new building (from 15 June 2009 to 31 July 2009); Period 3, the period of natural ventilation along with bake-out (from 1 August 2009 to 17 October 2009); and Period 4, an approximately 6 month period after bake-out (from 18 October 2009 to 30 April 2010).

The teacher in the day-care center was instructed to monitor pruritus levels and to record symptoms in a diary for each child with AD throughout the study period. The level of pruritus was measured using a Likert-type scale of 0 to 10 on a daily basis.

This study was approved by the institutional review board of Samsung Medical Center. Informed consent was obtained from the parents of all participating children.

### Measurement of indoor and outdoor air pollutant levels

Outdoor air quality data were obtained from the closest air quality monitoring station which was operated by the Seoul Metropolitan Government Research Institute of Public Health and Environment. The day-care center and the monitoring station were 1.9 km apart. Indoor air quality was monitored in the center of the second floor of the day-care center throughout the study period. Indoor air pollutant levels were measured according to the same model used in the air quality monitoring station.

Indoor and outdoor air pollutants measured in this study were nitrogen oxides (NO, NO_2_, and NO_x_), particulate matter (PM_10_, PM_2.5_, and PM_1.0_), total volatile organic compounds (TVOC), and 56 species of VOCs including benzene, toluene, ethyl-benzene, xylene, and styrene. The measurement of NO_2_ was performed with a NO_x_ analyzer (NA-623, Kimoto, Osaka, Japan) using the chemical luminescent method. PM was measured by aerosol dust monitors (Grimm 107/165, Grimm Aerosol Technik, Ainring, Germany) using the light scattering method. VOCs were measured using an air server and thermal desorption device (ATD-400, Markes. UK). Fifty-six species of VOCs were continuously measured with a gas chromatography (GC) / flame ionization detector (FID) (CP-3800, Varian, Santa Clara, CA, USA). VOCs with a low molecular weight were separated by an alumina plot column, while those with a higher molecular weight were separated by a BP-1 column. Qualitative and quantitative analyses were performed with an FID. All pollutants were continuously measured 24 hours a day over the entire study period. We calculated hourly average levels for each pollutant and used the mean value for the 8 hours during the day when the study participants were at the day-care center.

### Statistical analysis

Comparison of indoor air quality between each period was conducted through analysis of variance (ANOVA). A comparison of the symptom scores of AD was performed using the chi-square test. To assess the effect of indoor air quality on AD, a generalized linear mixed model (GLMM) was used, with AD symptoms as the dependent variable, and indoor air quality as the independent variable. GLMM was applied because the observation data recorded in the diary represented individual colony data with self-correlation and the dependent variable was a binomial variable. To dichotomize the AD symptoms, diary scores greater than 2 on the Likert scale were classified as a positive symptom. The model specifications we used are as follows: Log *E(Y)* = *β*_0_ + *β*_1_ (pollutant) + *β*_2_ (temperature) + *β*_3_ (relative humidity) + *β*_4_ (season) + *β*_5_ (SCORAD at initial visit) + *β*_6_ (age) + *γ*_1_ (children) + *γ*_2_ (date), where *E(Y)* is the expected presence of atopic dermatitis symptoms; γ_1_ is the random effect; and γ_2_ is the random effect within a child. The time (date) effect for each child is a residual effect with a first-order autoregressive covariance structure, using the residual. This is covariance matrix of autoregressive(1): cov[ε_*i*_, ε_*j*_] = σ^2^ ρ^|i^*^- j^*^|^, the value i* and j* are derived for the *i*th and *j*th observations and are not necessarily the observation numbers. Considering the lag effects of indoor air pollutants, a moving average was used to represent the effect of indoor air quality on AD symptoms 2 days later. Relative risk (RR) was calculated using the regression coefficient, then the percent change of risk was calculated. The percent change of risk was defined as the percent change of AD symptoms (pruritus) increased by one unit for the specific pollutant (e.g., 1 ppb increase in toluene) after controlling for confounding factors such as temperature, humidity, and season. A *P* value less than 0.05 was considered as statistically significant, and a *P* value less than 0.1 was regarded as borderline significant. SAS version 9.1 (SAS Institute, Inc., Cary, NC, USA) was used for all statistical analyses.

## Results

**Fig 1:**
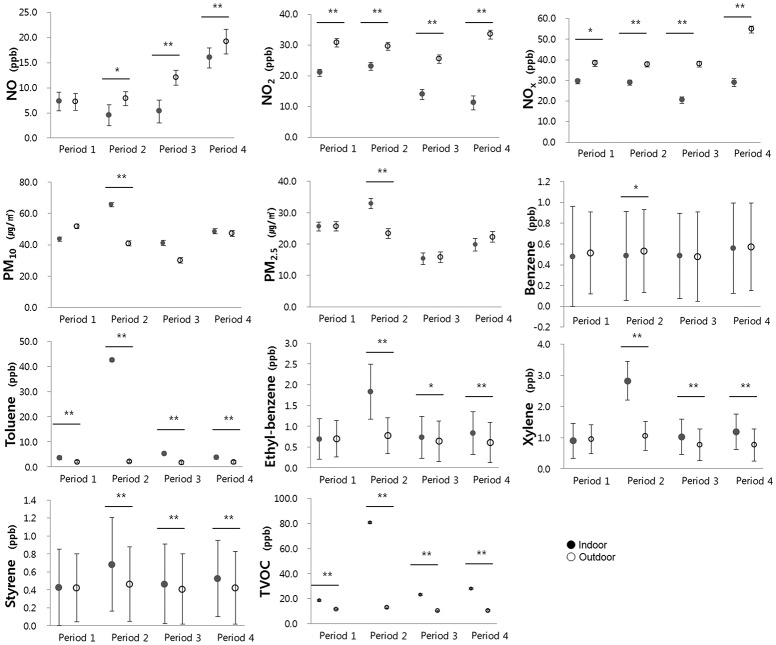
Indoor and outdoor air pollutant levels according to the study period. Circles and bars represent geometric mean ± geometric standard deviation. *P < 0.05, ** *P* < 0.01

In order to find whether air pollutants in a new building originate from sources indoors, we observed indoor air pollutant levels according to each period and compared these levels with outdoor air pollutant levels (Fig. 1).

Indoor levels of nitrogen oxides (NO, NO_2_, NO_X_) were lower than outdoor levels throughout the study period. In contrast, the concentrations of the other indoor pollutants such as particulate matters and VOCs were mostly higher than outdoors. In particular, the indoor concentrations of PM_10_, PM_2.5_, toluene, ethylbenzene, xylene, styrene, and TVOC were substantially higher during Period 2 compared with the outdoors. These results raised the possibility that the pollutants came from sources indoors and were related to the new building.

Table 1 shows the levels of indoor air pollutants, temperature and humidity in the day-care center over the study period.

**Table 1:** Levels of indoor air pollutants, temperature and humidity over the study period.

	Standard	Period 1	Period 2	Period 3	Period 4
Temperature (°C)		25.6±1.3	26.1±0.9	25.8±1.3	22.5±1.9
Humidity (%)		50.3±9.1	60.4±6.8	49.6±9.0	32.8±7.9
PM_10_ (μm/m^3^)	100	43.7±1.4	65.6±1.3	41.1±1.6	48.5±1.6
PM_2.5_ (μm/m^3^)		25.6±1.4	32.9±1.6	15.3±1.9	19.8±2.0
PM_1.0_ (μm/m^3^)		20.9±1.4	27.4±1.8	11.2±2.2	15.9±2.2
NO (ppb)		7.3±1.9	4.5±2.1	5.3±2.3	16.0±2.0
NO_2_ (ppb)	50	21.0±1.2	23.1±1.3	13.9±1.7	11.2±2.3
NOx (ppb)		29.5±1.2	28.9±1.2	20.5±1.6	28.9±1.9
Benzene (ppb)		0.5±0.5	0.5±0.4	0.5±0.4	0.6±0.4
Toluene (ppb)		3.6±0.7	42.5±0.7	5.3±0.7	3.8±0.7
Ethyl-benzene (ppb)		0.7±0.5	1.8±0.7	0.7±0.5	0.8±0.5
Xylene (ppb)		0.9±0.6	2.8±0.6	1.0±0.6	1.2±0.6
Styrene (ppb)		0.4±0.4	0.7±0.5	0.5±0.4	0.5±0.4
TVOC (ppb)	400	18.5±0.6	80.5±0.7	23.2±0.6	28.0±0.6

Standard levels for indoor air quality control in public use facilities are set by the Ministry of Environment, Republic of Korea.

All levels are written as geometric mean ± geometric standard deviation.

Periods 1 to 4 are described in the methods section.

PM_10_, particles < 10 μm in diameter; PM_2.5_, particles < 2.5 μm in diameter; PM_1.0_, particles < 1.0 μm in diameter; NO, nitrogen oxide; NO_2_, nitrogen dioxide; NO_X_, nitrogen oxide compounds; TVOC, total volatile organic compound; ppb, parts per billion.

Each level represents the geometric mean value for 8 hours during the day (08:00 to 16:00) when study participants were at the day-care center. For example, the TVOC concentration during Period 1 was 18.5 ppb, rose up to 80.5 ppb during Period 2, and declined to 23.2 and 28.0 ppb during Periods 3 and 4, respectively. Indoor concentration of particulate matters increased in Period 2, but fell to a level similar to the outdoor concentration after Period 3. These results demonstrated that the increased concentration of indoor air pollutants during Period 2 was reduced by natural ventilation along with bake-out during Period 3. In particular, the concentrations of toluene and TVOC decreased by 88% and 71%, respectively, between Periods 2 and 3. The level of NO_2_ during the study period, ranging from 1.1 to 43.1 ppb, was maintained at a concentration below the recommended value (50 ppb) for indoor air quality control in Korea. However, the maximum level of PM_10_ and TVOC exceeded the primary standard level for a short period of time, reaching up to 114.5 μg/m^3^ and 646.6 ppb, respectively.

The rates of positive AD symptoms for each period were compared to look at the relationship between indoor air pollution and AD symptoms. From symptom records of 1,428 person-days, 137 were written in Period 1, 157 in Period 2, 378 in Period 3, and 756 in Period 4 (Table 2).

**Table 2:** Positivity rate for atopic dermatitis symptom by study period.

	Period 1	Period 2	Period 3	Period 4
Days observed	20	24	56	137
Number of records (person-days)	99	157	378	756
Mean daily symptom positive rate (%)*	31.9 b^†^	43.8 a	50.5 a	37.0 b

Periods 1 to 4 are described in the methods section.

* *P*<0.0001 by comparison between each period.

^†^ *P*<0.05 by Duncan's post hoc multiple comparison.

After moving into the new building, the rate of positive AD symptoms increased from 31.9% in Period 1 to 43.8% in Period 2. This rate maintained at 50.5% in Period 3, suggesting that there was a sustained effect on AD symptoms despite a dramatic decrease in indoor air pollutant levels after natural ventilation and bake-out. In Period 4, the rate of positive AD symptoms dropped to 37.0% (*P* < 0.0001). When analyzed by Duncan's post hoc multiple comparison, the difference in AD symptom-positive rates was statistically significant between each period (*P* < 0.05).

The effect of indoor air quality on AD symptoms was evaluated in an unlagged and lagged model (Table 3), since skin symptoms could be delayed after exposure to air pollutants.

**Table 3:** The effect of indoor air pollutants on pruritus in patients with atopic dermatitis in unlagged and lagged models.

Indoor air pollutant^†^	Unlagged model				Lagged model*			
	% change of risk	95% CI		*P* value	% change of risk	95% CI		*P* value
PM_10_ (μm/m^3^)	0.17	-0.11	0.46	0.236	0.24	-0.18	0.66	0.264
PM_2.5_ (μm/m^3^)	-0.01	-0.42	0.39	0.946	0.04	-0.58	0.67	0.899
PM_1.0_ (μm/m^3^)	-0.04	-0.46	0.39	0.857	0.04	-0.62	0.71	0.899
NO (ppb)	0.24	-0.50	0.99	0.518	0.21	-0.76	1.18	0.678
NO_2_ (ppb)	-0.47	-1.44	0.51	0.347	-1.08	-2.50	0.36	0.140
NOx (ppb)	-0.01	-0.54	0.52	0.960	-0.18	-0.93	0.58	0.641
Benzene (ppb)	12.00	-0.93	26.62	0.070	5.94	-11.62	26.99	0.533
Toluene (ppb)	7.78	-1.19	17.57	0.091	12.73	-0.01	27.09	0.050
Ethyl-benzene (ppb)	4.29	-5.08	14.58	0.382	1.87	-9.82	15.07	0.766
Xylene (ppb)	2.68	-1.83	7.40	0.249	0.74	-5.05	6.88	0.807
Styrene (ppb)	2.95	-4.94	11.49	0.475	-2.47	-12.97	9.30	0.667
TVOC (ppb)	0.03	-0.25	0.31	0.843	0.00	-0.38	0.39	0.982

Percent change of risk and 95% CI were calculated by using a regression coefficient (β) and the following equation: percent change of risk = (exp[β]- 1) × 100 and 95% CI = (exp[β]- 1 ± 1.96 SE). Percent change of risk indicates a change in AD symptoms according to an increase of 1 unit of each pollutant.

PM_10_, particles < 10μm in diameter; PM_2.5_, particles < 2.5μm in diameter; PM_1.0_, particles < 1.0μm in diameter; NO, nitrogen oxide; NO_2_, nitrogen dioxide; NO_X_, nitrogen oxide compounds; TVOC, total volatile organic compound; ppb, parts per billion.

*In lagged model, a moving average was used to evaluate the lag effect of indoor air quality on symptoms of atopic dermatitis 2 days later.

^†^Adjusted by temperature, humidity, season, SCORAD at initial visit, and age.

In the unlagged model, we analyzed the relationship between indoor air pollutant levels and AD symptoms on the same day. An increased concentration of benzene by 1 ppb was associated with a 12.0% (95% CI: -0.9 to 26.6) increase in AD symptoms, although statistical significance was borderline (*P* = 0.07). When the delayed effect of indoor air pollutants on AD symptoms 2 days later was evaluated, AD symptoms significantly increased by 12.7% (95% CI: -0.01 to 27.1) as indoor levels of toluene increased by 1 ppb (*P* = 0.05).

## Discussion

Indoor air pollutants, including formaldehyde and VOCs, are emitted from newly constructed or decorated buildings [2]. In the present study, we used a prospective, longitudinal study, not a cross-sectional case-control study, to investigate the clinical effects of indoor air pollutants on AD symptoms in a newly-built day-care center. We found that itching score was correlated with levels of chemicals such as toluene which increased after moving into a new building. Clinically relevant improvement was demonstrated after the concentrations of these chemicals were reduced by bake-out and ventilation. Our results suggest that indoor air pollution act as a risk factor for aggravation of AD. A previous study showed that short-term exposure to formaldehyde and NO_2_ for 4 hours increased transepidermal water loss (TEWL) in children with AD [20]. In a total body exposure chamber, VOCs damaged the skin barrier and this effect was enhanced by prior exposure to house dust mite allergen (Der p 1) in patients with AD [21]. In an epidemiological study, redecoration activities such as painting, floor covering and new furniture before birth and in the first year of life seemed to be associated with the risk of eczema [22]. Our findings support the fact that indoor air pollution contributes to worsening of skin symptoms in children with AD.

Toluene is one of a diverse group of chemicals known as VOC and mostly comes from indoor sources like paints and coatings. It is commonly added to gasoline, and can therefore enter the home as motor vehicle exhaust. In our study, an increased concentration of toluene by 1 ppb was associated with a 12.73% increase in AD symptoms 2 days later. Although outdoor toluene did not show a delayed effect on AD symptoms in our previous study [23], it must be considered that the extent of exposure to toluene was very different. The geometric mean value of indoor toluene level during Period 2 of the present study was 42.5 ppb, whereas the concentration of outdoor toluene in the previous study was approximately 4 ppb. Benzene in indoor air originates from outdoor air and also from sources indoors such as building materials and furniture. Our study demonstrated that the indoor benzene level was weakly associated with AD symptoms on the same day, but this effect disappeared 2 days later. This result is consistent with our previous study in which elevated concentration of outdoor benzene are associated with an increase in AD symptoms on the following day (lag 1) without delayed effect 2 days later (lag 2) [23]. However, it must be taken into account that indoor levels of benzene did not change over the study period. These results indicate that the association was not related with moving into a new building.

The indoor air quality in day-care centers in Korea is regulated by the Indoor Air Quality Control in Public Use Facilities Act of the Ministry of Environment. The primary standard levels for PM_10_, NO_2_ and TVOC are 100 μm/m^3^, 50 ppb and 400 ppb, respectively. Of note, our patients showed symptom aggravation, although most pollutants were maintained at concentrations below primary standards during the study period. Since the standard level is determined to reduce health risk in a general healthy population, our data suggest that children with AD might be more vulnerable to indoor air pollutants. Although threshold levels for eliciting skin symptoms in AD are not known, more effort to reduce indoor air pollutants below the standard level in public use facilities seems to be needed in case of children with AD.

The exact mechanism through which indoor air pollutants aggravate AD symptoms remains unclear. Repeated exposure of toluene, xylene, and formaldehyde onto mouse ears induced ear swelling and increased the expression of interleukin (IL)-4, brain-derived neurotrophic factor (BDNF), neutrotrophin-3, and transient receptor potential vanilloid-1 (TRPV-1) mRNAs in the ears [24]. Acute microvascular leakage was produced by topical application of VOCs to rat skin, and the reaction was related to the release of tachykinins [25]. In addition, dermal application of formaldehyde results in the expression of IL-4, IL-13, and IFN-γ mRNAs in mouse spleen and lymph nodes [26]. Indoor air pollutants might lead to skin inflammation and aggravation of symptoms in patients with AD through a similar mechanism, although inflammatory mediators were not evaluated in the present study.

Our results showed that indoor air quality improved significantly during the period of natural ventilation and bake-out compared with the initial moving-in period. Bake-out is a process to heat the house for quickly evaporating VOCs followed by ventilation to release the pollutants outside. A previous study reported a 50% decrease in formaldehyde and toluene concentrations after the bake-out procedure in a newly-built apartment with a heating system [27]. Similarly, our study revealed that bake-out effectively reduced the concentrations of toluene and TVOC. This process can be used for preventing aggravation of AD when patients move into a newly-built house.

In the present study, we found that indoor air pollution is an important contributor to AD symptoms. The strengths of our study are a longitudinal study design for a long-term period and continuous measurement of indoor air pollutant levels to overcome the issue of single time measurement. However, there might be other indoor air pollutants that more strongly affect AD or act as an adjuvant. One example is phthalate, which is classified as semi-volatile organic compounds (SVOCs). Actually, there have been case-control studies in which some phthalates in dust are associated with eczema and wheezing [16,28,29]. Further studies are needed to investigate the relationship between allergic diseases and various indoor air pollutants such as SVOCs. A limitation of our investigation was that we used a teacher-reported itching score, not an objective assessment by a physician, as the dependent variable. However, only one teacher participated in the study and evaluated the change in symptom scores. This maintained assessment consistency and eliminated inter-observer variability. In addition, we did not measure the level of formaldehyde, one of the important indoor air pollutants, found in building materials and home furnishings. This was because we used the same measuring instrument as in the air quality monitoring station. Nevertheless, toluene significantly aggravated AD symptoms. Formaldehyde should be evaluated in future studies.

In conclusion, indoor air pollutants increase the risk of AD aggravation in children and toluene in the indoor environment might act as an aggravating factor.
